# Microstructures and Mechanical Properties of Co-Cr Dental Alloys Fabricated by Three CAD/CAM-Based Processing Techniques

**DOI:** 10.3390/ma9070596

**Published:** 2016-07-20

**Authors:** Hae Ri Kim, Seong-Ho Jang, Young Kyung Kim, Jun Sik Son, Bong Ki Min, Kyo-Han Kim, Tae-Yub Kwon

**Affiliations:** 1 Department of Dental Science, Graduate School, Kyungpook National University, Daegu 700-412, Korea; harry@knu.ac.kr (H.R.K.); moodttl@naver.com (S.-H.J.); 2 Department of Conservative Dentistry, School of Dentistry, Kyungpook National University, Daegu 700-412, Korea; wisekim@knu.ac.kr; 3 Korea Textile Development Institute, Daegu 703-712, Korea; sonjk1@empas.com; 4 Center for Research Facilities, Yeungnam University, Gyeongsan 712-749, Korea; 5 Department of Dental Biomaterials, School of Dentistry, Kyungpook National University, Daegu 700-412, Korea; kyohan@knu.ac.kr

**Keywords:** Co-Cr alloy, fabrication techniques, CAD/CAM, microstructure, mechanical properties

## Abstract

The microstructures and mechanical properties of cobalt-chromium (Co-Cr) alloys produced by three CAD/CAM-based processing techniques were investigated in comparison with those produced by the traditional casting technique. Four groups of disc- (microstructures) or dumbbell- (mechanical properties) specimens made of Co-Cr alloys were prepared using casting (CS), milling (ML), selective laser melting (SLM), and milling/post-sintering (ML/PS). For each technique, the corresponding commercial alloy material was used. The microstructures of the specimens were evaluated via X-ray diffractometry, optical and scanning electron microscopy with energy-dispersive X-ray spectroscopy, and electron backscattered diffraction pattern analysis. The mechanical properties were evaluated using a tensile test according to ISO 22674 (*n* = 6). The microstructure of the alloys was strongly influenced by the manufacturing processes. Overall, the SLM group showed superior mechanical properties, the ML/PS group being nearly comparable. The mechanical properties of the ML group were inferior to those of the CS group. The microstructures and mechanical properties of Co-Cr alloys were greatly dependent on the manufacturing technique as well as the chemical composition. The SLM and ML/PS techniques may be considered promising alternatives to the Co-Cr alloy casting process.

## 1. Introduction

The production of metallic restorations in the dental laboratory has conventionally been carried out using the traditional cast method based on the lost wax process [1,2,3]. Although the alloys of choice were gold-based when the casting technique was first developed, they were gradually replaced by base-metal alloys such as nickel-chromium (Ni-Cr) and cobalt-chromium (Co-Cr) alloys [2,4]. Ni-Cr alloys containing beryllium (Be), frequently used in the past, are no longer recommended because of allergic reactions and potential carcinogenic effects of Ni and Be [5,6]. Co-Cr alloys are the most common base-metal alternative for patients known to be allergic to nickel [4,5,7]. They are relatively inexpensive compared to noble alloys and exhibit material properties considered suitable for dental reconstructions, such as high strength, high modulus of elasticity, and high corrosion resistance [4,5]. However, the Co-Cr alloys have the highest melting ranges of the casting alloys, with the exception of titanium alloys, making it difficult to manipulate these alloys in the dental laboratory [5,8]. Additionally, their high hardness and low ductility makes them difficult to finish and polish [4,8].

In the last decade, novel manufacturing processes using computer-aided design/computer-aided manufacturing (CAD/CAM) technologies are becoming increasingly important for the production of biomedical devices and dental prostheses [9,10,11]. Co-Cr dental restorations can be fabricated using either of the two main approaches based on CAD/CAM processing: subtractive manufacturing and additive manufacturing [6,7]. In CAD/CAM milling, one of the subtractive processes for producing metallic restorations [12], the formation of casting-induced flaws and porosities may be minimized by using Co-Cr alloy blanks manufactured under standardized industrial conditions [4]. However, increased tool and machine wear caused by the high rigidity of the “solid” blank [3,8] and high acquisition and maintenance costs are the main disadvantages of the technique [6].

Additive manufacturing assisted by CAD/CAM technology is an alternative method for producing metallic restorations. Selective laser melting (SLM) is an additive manufacturing procedure that produces metal components directly from a three-dimensional (3D) CAD model by fusing fine layers of metal powder using a high-power focused laser beam [13]. However, this process currently requires very expensive equipment and is, thus, restricted to large CAD/CAM centers in the dental industry [3].

Another recent development in the production of Co-Cr dental restorations is dry milling of “soft” Co-Cr blanks, in which the alloy powder is finely distributed in a binder material that is capable of burn-out, thereby reducing manufacturing time and costs [3,6]. The milled reconstruction must subsequently be sintered to full density in a special, high-temperature sintering furnace under an argon protective gas atmosphere at approximately 1300 °C [6]. As the processing steps are quite similar to those of pre-sintered zirconia [6], the metallic restorations can be extensively applied in ordinary dental laboratories using available CAD/CAM equipment [3,11].

Co-Cr alloy microstructures are strongly associated with alterations in chemical composition and mechanical properties which vary according to manufacturing technique [12]. It has been recently reported that Co-Cr dental alloys fabricated by SLM showed different microstructures and, thus, properties from those produced by conventional casting [13,14,15]. In contrast, limited information is available regarding the microstructural characteristics and mechanical properties of Co-Cr dental alloys prepared by milling, particularly using newly developed milling/post-sintering techniques [3,7,11]. Information on the comparative mechanical properties of Co-Cr alloys fabricated using CAD/CAM-based processing techniques and the relationships of such properties with alloy microstructures will enable the dental clinician to select appropriate alloys in different clinical situations where metal restorations are indicated [9].

The purpose of this in vitro study was to investigate the microstructural characteristics and mechanical properties of Co-Cr alloys fabricated using three CAD/CAM-based processing techniques (milling, SLM, and milling/post-sintering) and to compare them to those of cast Co-Cr alloy. The brand names, manufacturing methods (group codes), manufacturers, and compositions of the four Co-Cr alloys used are summarized in Table 1. The null hypothesis was that there would be similar microstructures and mechanical properties among the groups prepared with different manufacturing techniques.

## 2. Results

### 2.1. X-ray Diffraction Analysis

**Figure 1:**
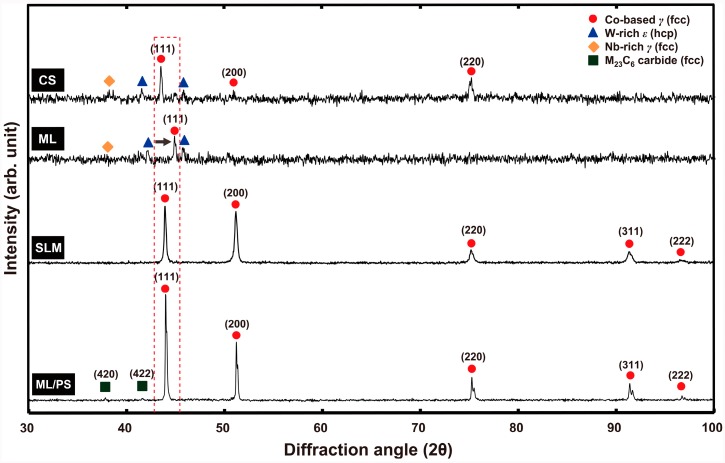
XRD patterns of the Co-Cr alloys tested. The black arrow in the ML group indicates a shift of the (111) plane. The SLM group showed only the presence of the Co-based *γ* (fcc) phase. The carbide (M_23_C_6_) formation was identified only in the ML/PS group.

The X-ray diffraction (XRD) patterns of the four Co-Cr alloys tested are presented in Figure 1. In all groups, the Co-based *γ* (face-centered cubic, fcc) matrix phase with a nominal parameter a = 0.35447 nm (ICDD card no. 15-806) was identified. In the casting (CS) and milling (ML) groups, two intermetallic phases, assumed to be tungsten (W)-rich *ε* (hexagonal close-packed, hcp) and niobium (Nb)-rich *γ* (fcc) phases, were detected, together with the matrix phase. The SLM group exhibited the single matrix phase. In the milling/post-sintering (ML/PS) group, peaks indexed as M_23_C_6_ (M = Cr, Co, Mo) metal carbides with a cubic structure (ICDD card no. 35-783) were detected.

### 2.2. Microscopic Characterization

**Figure 2:**
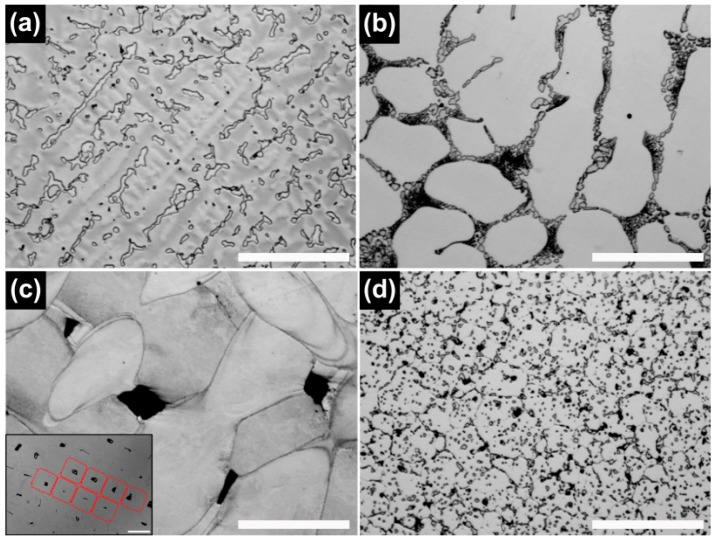
Optical microscopy images of the Co-Cr alloys tested. (**a**) CS; (**b**) ML; (**c**) SLM; and (**d**) ML/PS (200×, scale bar = 100 μm). The red rounded squares in the small box in C indicate laser scan traces (small box in C: 100×, scale bar = 100 μm).

**Figure 3:**
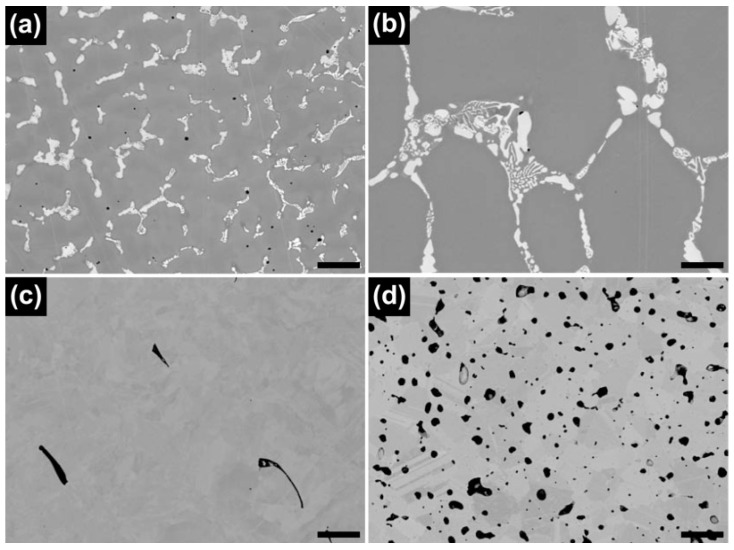
BSE images of the Co-Cr alloys tested. (**a**) CS; (**b**) ML; (**c**) SLM; and (**d**) ML/PS (500×, scale bar = 30 μm). The secondary phases were observed in the CS and ML specimens, but such phases were absent in the SLM specimen.

Figure 2 and Figure 3 show the optical microscopy (OM) and backscattered electron (BSE) images of the Co-Cr alloy specimens prepared using the four different manufacturing techniques. In both the CS and ML groups, the typical dendritic grains predominated and the intermetallic phases were precipitated along the grain boundaries, but the ML group showed a substantially larger grain size than the CS group. The SLM group clearly exhibited the laser scan traces (the small box in Figure 2c). In the BSE images of the SLM and ML/PS groups, the presence of fine grains was recognized by a weak orientation contrast (Figure 3c,d). The BSE image of the ML/PS group also revealed crystal twinning.

**Figure 4:**
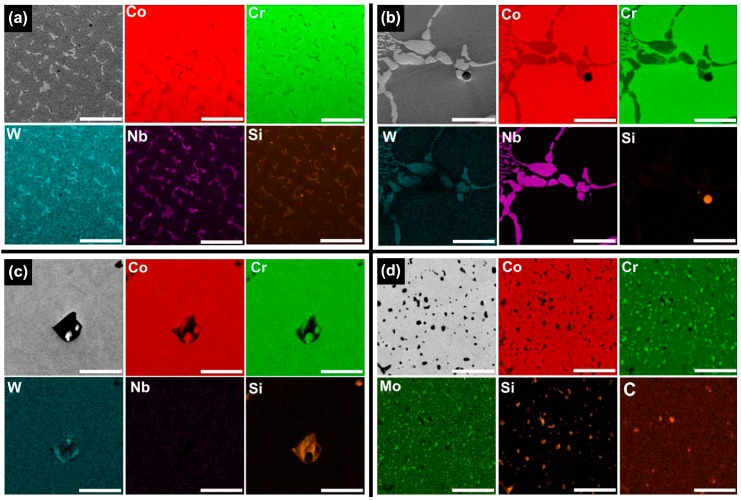
BSE and corresponding EDS mapping images for each group. (**a**) CS; (**b**) ML; (**c**) SLM; and (**d**) ML/PS (500×, scale bar = 50 μm).

The energy dispersive X-ray spectroscopy (EDS) mapping images of the Co-Cr alloys tested, shown in Figure 4, more clearly reveal the presence of the matrix, intermetallic, and metal carbide phases for each group. The images also show silicon (Si)-rich inclusions in all the groups. The elemental distribution of carbon was detected only in the ML/PS group.

**Figure 5:**
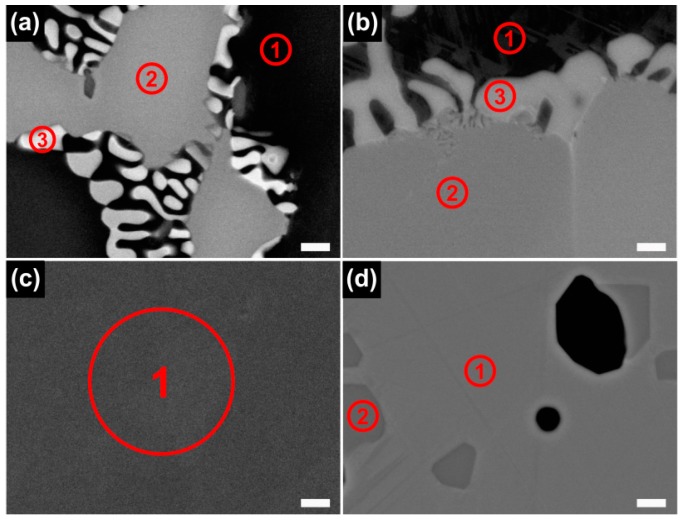
BSE images of the Co-Cr alloys for EDS point analysis. (**a**) CS; (**b**) ML; (**c**) SLM; and (**d**) ML/PS (10,000×, scale bar = 1 μm). In each figure, the numbers indicate the points subjected to the EDS analysis.

Figure 5 and Table 2 present the results of the EDS point analysis for each alloy tested. In the CS and ML groups, the darker area (point 1) was found to be the Co-based matrix phase, whereas the brighter regions were assumed to be rich in the heavier available elements, primarily W (point 2) and Nb (point 3). On the other hand, such contrast was not identified for the SLM specimen. The ML/PS group showed the Co-based matrix phase and the dispersed phase, precipitates being identified by the dark gray contrast relative to the matrix, indicating carbide formation.

**Figure 6:**
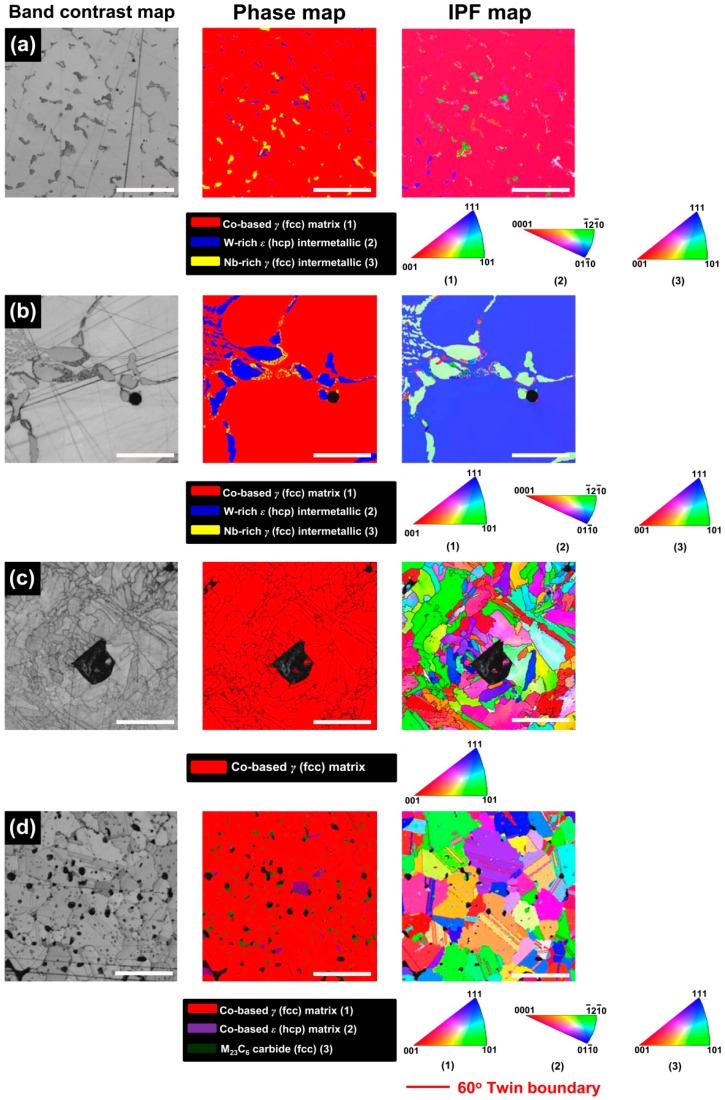
BSE image (band contrast map) for each group and corresponding phase and inverse pole figure (IPF) maps. (**a**) CS; (**b**) ML; (**c**) SLM; and (**d**) ML/PS (500×, scale bar = 50 μm).

The electron backscattered diffraction (EBSD) images and corresponding phase maps and inverse pole figure (IPF) maps of the alloys tested are given in Figure 6. The results were generally consistent with those of XRD analysis (Figure 1). In the ML/PS group, however, the EBSD analysis newly revealed the presence of a small portion of a Co-based *ε* (hcp) phase (purple in the phase map), together with a Co-based *γ* (fcc) phase (Figure 6d). In addition, the IPF map revealed that the ML/PS group had a significant number of annealing twins inside the matrix phase, as seen in the BSE image (Figure 3d). When the grain size for each group was measured on corresponding IPF maps, the ML group showed a substantially larger grain size (ca. 4000 μm) than the CS group (ca. 1500 μm). The SLM and ML/PS groups showed similar grain size (ca. 30 vs. 35 μm, respectively).

### 2.3. Mechanical Properties and Fracture Surfaces

**Figure 7:**
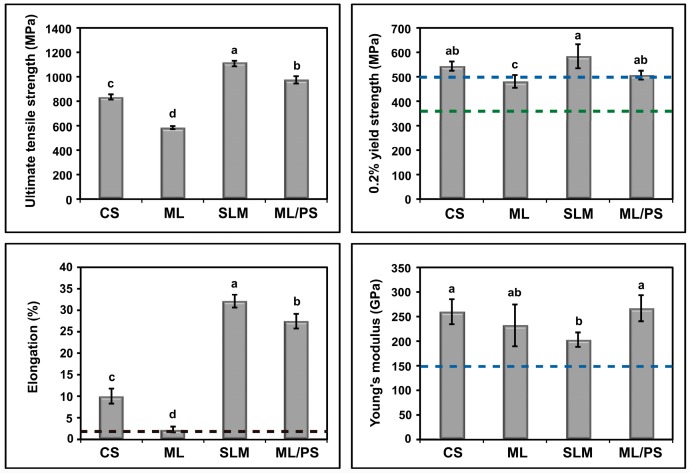
Comparison of mechanical properties of the four Co-Cr alloys tested (*n* = 6). For each figure, means with different letters indicate statistical differences between groups (*p* < 0.05). The green and blue horizontal lines indicate the type 4 and 5 criteria (minimum value), respectively, in ISO 22674. The type 4 and 5 criteria are the same for percent elongation (black horizontal line).

Figure 7 shows the mechanical properties of the Co-Cr alloy specimens prepared using the four different manufacturing techniques. The SLM group showed the highest mean ultimate tensile strength followed by the ML/PS, CS, and ML groups. The four groups can be arranged in decreasing order of mean yield strengths (in MPa) as follows: SLM (580 ± 50), CS (540 ± 20), ML/PS (510 ± 20), and ML (480 ± 20). The mean percent elongation values were substantially higher in the SLM (32 ± 2) and ML/PS (27 ± 2) groups than the CS (10 ± 2) and ML (2.3 ± 0.7) groups. Although the ML/PS group showed the highest mean Young’s modulus (270 ± 30 GPa) followed by the CS (260 ± 20 GPa), ML (230 ± 40 GPa), and SLM (200 ± 10 GPa) groups, the values for all the groups were above 150 GPa.

**Figure 8:**
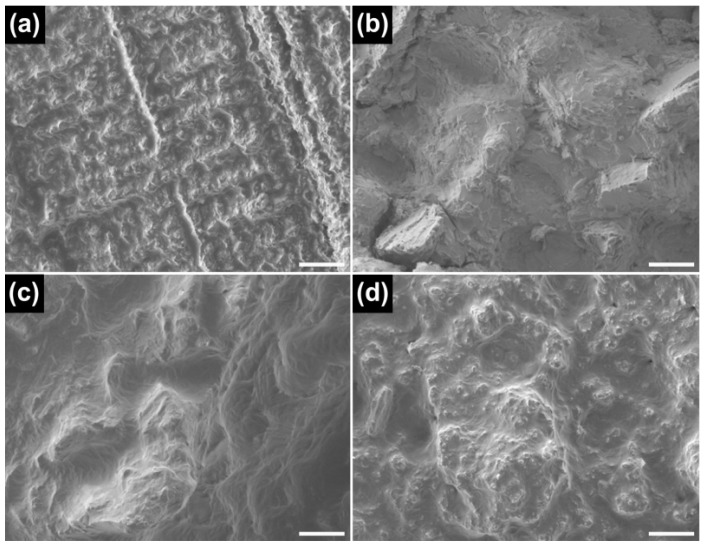
SEM images of the fractured surfaces after tensile test. (**a**) CS; (**b**) ML; (**c**) SLM; and (**d**) ML/PS (500×, scale bar = 30 μm).

The representative SEM images of the fractured surfaces after the tensile test are shown in Figure 8. The CS and ML groups showed deep furrows randomly distributed in the brittle cleavage, indicating a brittle fracture pattern (Figure 8a,b). In contrast, the fractured surfaces of the SLM and ML/PS groups possessed lacerated ridges and dimples (mixed-rupture characteristics of quasi-cleavage) which attest to ductile tearing (Figure 8c,d).

## 3. Discussion

In the present in vitro study, the microstructures and mechanical properties of Co-Cr dental alloys fabricated by four different manufacturing methods (casting and three CAD/CAM-based processing techniques) were investigated and compared to each other. The findings of this study clearly showed that the microstructures, and, thus, mechanical properties of the alloys, were greatly dependent on the manufacturing techniques, together with the chemical compositions of the alloys used. Thus, the null hypothesis that there would be similar microstructures and mechanical properties among the groups prepared by different manufacturing techniques was rejected. As shown in Figure 7, the mechanical properties of all the alloys generally satisfied the type 5 criteria in ISO 22674 (0.2% yield strength: >500 MPa; elongation: >2%; Young’s modulus: >150 GPa) [16]. However, the ML specimens showed a mean yield strength value less than 500 MPa (480 MPa) and, thus, satisfied the type 4 criteria (0.2% yield strength: >360 MPa) [16]. Thus, all of the alloys tested in this study could be used for the construction of the type 4 or 5 dental appliances, such as removable partial dentures, clasps, thin veneered crowns, and wide-span bridges, in terms of mechanical properties [16].

It is known that Co undergoes an allotropic phase transformation from a high temperature *γ* (fcc) phase to the low temperature *ε* (hcp) phase [1,17,18]. Co-Cr alloys exhibit a dendritic *γ* (fcc) metastable matrix because the unstable fcc structure is retained at room temperature due to the low rate of the fcc → hcp transformation [7,19]. The XRD and EBSD analyses (Figure 1 and Figure 6) indicated that the microstructures of all the tested groups consisted of the *γ* (fcc) matrix phase, which mainly comprised Co and Cr [7]. The retained unstable fcc structure is believed to be associated with some characteristic properties of Co-based alloys, such as high yield strength, high work-hardening rates, limited fatigue damage under cyclic stresses, and the ability to absorb stresses (through transformation of fcc to hcp structure) [5].

Although all of the tested groups consisted of Co-based *γ* (fcc) matrix phase, the OM and BSE images demonstrated great microstructural differences among the groups (Figure 2 and Figure 3). The CS and ML groups showed the typical cast structure of a Co-Cr alloy, being composed of a matrix and a heavier dispersed phase that occupy the grain boundaries [13]. The dispersed phase was expected to be rich in the heavier available elements, primarily W and Nb (Table 1), and this was confirmed by the XRD and EDS analysis (Figure 1, Figure 5 and Table 2). W or Mo is added to Co-Cr alloys to achieve a finer grain structure, thereby enhancing the mechanical properties [20]. This favorable effect is diminished when W or Mo is segregated in the intermetallic compounds rather than being dispersed within the matrix by removing W or Mo from the solid solution [7]. A slight shift of the (111) plane was observed only in the ML group (black arrow in Figure 1), reflecting decreased cell size due to a low concentration of heavy metal elements (W or Mo) in the Co-based alloy [21].

The microstructures of milled Co-Cr alloy depend on the initial quality of the pre-manufactured metallic block [13,17]. The ML group showed inferior mechanical properties to the CS group (Figure 7), in particular a low elongation value (mean 2.3%). This may be primarily due to substantially larger grain size in the ML group than in the CS group. A fine grain structure is generally more desirable because it ensures uniform properties of the alloy, thereby increasing hardness and yield strength of the alloy [22,23]. Moreover, the ML group showed a coarser distribution of the intermetallic phases than the CS group. The low elongation value of the ML group seems attributable to the increased brittleness of the alloy induced by the formation of “continuous” intermetallics along the interdendritic regions into the Co-based alloy matrix [12]. Moreover, W-rich intermetallic has the hcp phase, which is more brittle than the fcc phase. The fractured surface of the ML group after the tensile test showed a typical brittle fracture pattern (Figure 8). The findings (i.e., poor ductility) suggest that adjustments of clasps constructed with the ML alloy may potentially cause fracturing [23].

In contrast to the CS and ML groups, the SLM group definitely showed a single matrix phase (Figure 1) [7]. Dispersed phase was not identified by XRD analysis nor by BSE imaging, possibly because the rapid solidification of fused metallic particles led to a very fine phase size that was below the resolution of the analyses [7]. However, the EBSD analysis clearly revealed the presence of fine grain structures in the single matrix phase (Figure 6c). In the case of SLM technique, full local melting and rapid solidification minimizes the porosity and produces a homogeneous and dense material, with improved mechanical properties [13,24]. However, the development of porosity in SLM alloys seems strongly dependent on the proper adjustment of operating conditions, including laser power, scan spacing, scan rate, and scan thickness [13,14]. Although the black areas on the OM image (Figure 2c) may indicate some porosity formed by overheating directly under the laser beam center, the SLM specimens showed only slight porosity (Figure 3c) [25].

Overall, the SLM group showed superior mechanical properties to the other groups (Figure 7), in agreement with recent studies showing that SLM technique provides Co-Cr alloys with enhanced mechanical properties [7,14]. A high yield strength and relatively low but sufficient modulus of elasticity for the SLM group suggest that the alloy is appropriate for constructing both removable partial denture frameworks and clasps, with the advantage that the restorations can be made thinner in cross-section while maintaining adequate rigidity [2,5]. The high yield strength value also suggests that slow loosening of retention of Co-Cr clasps in service due to permanent deformation can be avoided by the use of the alloy [23]. In addition, the great percent elongation value (i.e., great ductility) indicates that adjustments of clasps produced from the SLM alloy can be made without fracturing [23].

For metallic restoration fabricated by SLM, the anisotropy of the mechanical properties in the builds may also merit close consideration. The substantially high elongation value of the SLM group (Figure 7) seems related to the building direction (in this study, the angle between the building and longitudinal direction was 0°). In a previous study [14], higher yield strength and lower elongation were observed when the dumbbell specimens were fabricated at 45° and 90°. This was confirmed by our preliminary tests (i.e., higher yield strength and lower elongation at 90° than at 0°). Nonetheless, these values still satisfied the type 5 criteria in ISO 22674, regardless of the direction of the builds [16]. In practical dental applications, however, the direction still should be considered to obtain a metallic restoration with more desirable mechanical properties.

The OM and BSE images showed that the ML/PS group had substantially smaller grain size than the ML groups (Figure 2 and Figure 3). The EBSD analysis revealed that the ML/PS group had two matrix phases, which comprised the *γ* (fcc) and *ε* (hcp) phases (Figure 6d). The *ε* (hcp) phase was not identified by the XRD analysis (Figure 1), probably due to its low phase content. The alloy did not show any intermetallic compound formation. Instead, the nucleation and growth of M_23_C_6_ carbide were identified in the grain boundary areas only in the ML/PS group (Figure 1, Figure 4, Figure 5 and Figure 6) [20]. Carbide precipitation at grain boundaries is the major strengthening mechanism of Co-Cr alloys. During crystallization, the carbides may become precipitated in the grain boundaries [23]. The fine precipitation of carbides can dramatically raise the strength and hardness of the alloy [2]. Discontinuous carbide formation at the grain boundaries is preferable to continuous carbide formation because it allows some slip and reduces brittleness [2,23].

The carbon content should be carefully controlled because even small changes in the carbon content can significantly alter the mechanical properties of the alloy [2]. Even though a raw Co-Cr alloy has a low carbon content, carbide formation may occur during the manufacturing process and, thus, affect the nominal properties of the produced alloy [7]. According to the manufacturer, the pre-sintered Co-Cr alloy blocks themselves do not contain carbon but do contain an organic binder (Table 1). During the sintering process, some of the organic binder may remain after most of it burns out. The high carbon content in the EDS point analysis (Table 2) seems to indicate residual carbon composition.

The EBSD map of the ML/PS group clearly confirmed the presence of twin boundaries (Figure 6d), shown in the BSE image by the orientation contrast (Figure 3d). Twin boundaries, a special kind of coherent boundary with low energy, contribute to many material properties, including interfacial energy, boundary diffusivity, and boundary mobility [26]. Twinning is frequently observed in many fcc or hcp metals when they are deformed at low temperatures and/or high strain rates, conditions that suppress dislocation motion [27]. Twin boundaries may be effective in blocking dislocation motions from one grain to the next by serving as barriers, similar to conventional grain boundaries [27,28,29]. It seems that the twin boundaries were formed to relieve stress during the fcc → hcp transformation immediately after post-sintering cooling, resultantly contributing to enhanced mechanical properties of the produced alloy [30].

The mechanical properties of the ML/PS group were relatively similar to those of the SLM group (Figure 7). Considering the substantially high ultimate tensile strength and elongation values of the ML/PS and SLM groups as compared to the CS and ML groups, the ML/PS and SLM alloys can be categorized as “tough” and highly ductile [12]. The two groups also showed a typical ductile fracture pattern after the tensile test (Figure 8c,d). Thus, the Co-Cr dental restorations fabricated by the ML/PS or SLM technique could be effectively burnished because of the high ductility of the alloy, notwithstanding the high yield strength [23].

Of the two different subtractive manufacturing methods, the ML/PS technique showed superior mechanical properties to the ML technique and nearly comparable ones to SLM technique (Figure 7). Thus, Co-Cr dental restorations can be relatively easily fabricated using a machine for milling of pre-sintered zirconia, without the need for very expensive technical equipment such as required for the SLM process, although a post-sintering step is mandatory [11]. However, the ML/PS alloy does not require post-manufacturing heat treatment. The post-sintering shrinkage of the ML/PS alloy should be carefully controlled during the CAD procedures in order to ensure high fitting accuracy of the restoration. Although these new techniques seem highly promising, many other properties, such as corrosion, metal-ceramic bonding, and biocompatibility, should be further investigated for more popular use in the dental field as cost-effective and reliable alternatives to the traditional casting technique [5].

It should be noted that the four Co-Cr alloys fabricated via four different manufacturing processes do not have exactly the same chemical compositions (Table 1) because a single alloy that could be used for the four manufacturing processes was not available [7]. The properties of the alloys may be affected not only by the principal elements but also by the minor alloying elements [12]. In addition, post-manufacturing heat treatment was not performed for all the test groups. Therefore, the differences in microstructures and mechanical properties cannot be attributable only to different processing techniques; this should be considered a limitation of this study [31].

The findings of this in vitro study suggest that the mechanical properties of Co-Cr-based alloys and the clinical behaviours of prosthetic restorations constructed with them are due to microstructures which can be altered via manufacturing techniques as well as the elemental compositions of the alloys. In addition, SLM and ML/PS techniques assisted by CAD/CAM technology may be considered promising alternatives to the traditional casting process in terms of mechanical properties. However, such newly-introduced technologies in the dental field present a wide spectrum of factors that should be tested and/or optimized to increase efficacy in the production of metallic dental restorations.

## 4. Materials and Methods

### 4.1. Specimen Preparation

To investigate the microstructures, disc-shaped Co-Cr alloy specimens (10 mm in diameter and 1 mm in thickness) were prepared using one of the four manufacturing processes: casting (CS group), milling (ML group), SLM (SLM group), and milling/post-sintering (ML/PS group). For each technique, the corresponding commercial Co-Cr alloy material was used. A disc-shaped 3D model designed with CAD software (AutoDesk Inventor, Autodesk, San Rafael, CA, USA) was used for the fabrication of all four types of specimens.

In the CS group, wax patterns (VisiJet^®^ M3 Dentcast, 3D Systems, Rock Hill, SC, USA) were prepared from the CAD data using a multi-material 3D Printer (ProJet^®^ 5500X, 3D Systems, Rock Hill, SC, USA). The patterns were embedded in a phosphate-bonded investment material (Univest Non-Precious, Shofu Inc., Kyoto, Japan), and then cast using the Co-Cr alloy (StarLoy C, DeguDent, Hanau-Wolfgang, Germany) with a centrifugal casting apparatus (Centrifico Casting Machine, Kerr Corp., Orange, CA, USA).

In the ML group, the CAD data was transmitted to a five-axis milling machine (Röders RXD5, Röders GmbH, Soltau, Germany) and the disc specimens were milled off a prefabricated Co-Cr alloy block (Magnum Lucens, Giacomo and C. S.N.C., Travagliato, Italy).

In the SLM group, the specimens were produced from the CAD data using a dental laser melting device (Concept Laser M1, Concept Laser GmbH, Lichtenfels, Germany). The laser power and laser scan speed were 100 W and 600 mm/s, respectively, for the contour (part border) scanning; power and scan speed were 160 W and 1100 mm/s, respectively, for the plane (part hatch (core)) scanning. The beam diameter was approximately 0.05 mm. The powder (Remanium^®^ star CL, Dentaurum GmbH and Co. KG, Ispringen, Germany) was applied to a stainless steel plate and laser-melted upwards in subsequent layers after a 30-μm-thick layer was completed until the final product was generated [7]. The axial (normal) direction of the disc-shaped specimens was fixed parallel to the building direction for each condition.

In the ML/PS group, a pre-sintered Co-Cr alloy block (Soft Metal™, LHK, Chilgok, Korea) was dry-milled using a milling machine (Zenotec T1, Wieland Dental + Technik GmbH and Co. KG, Pforzheim, Germany) to form disc-shaped specimens which were subsequently sintered to full density in a furnace (SinTagon, Denstar, Daegu, Korea) under the inert gas purge, using an argon (grade 4.0), at 1300 °C [6]. Post-sintering volume shrinkage of 11% (according to the manufacturer) was considered during the CAD design process.

The specimen surfaces for microstructure analyses were polished with wet silicon carbide paper (up to 2000-grit) and then with a 1 μm diamond suspension (Allied High Tech Products, Rancho Dominguez, CA, USA) on a polishing cloth using a rotary grinding/polishing machine (M-Prep 3, Allied High Tech Products). For optical microscopy analysis, the polished disc specimen surfaces were further electropolished in a solution of 5% H_2_SO_4_ and 95% CH_3_OH at 16 V at 273 K (0 °C) [14,32]. Specimens for electron backscatter diffraction (EBSD) analysis were finally polished using a 0.5 μm alumina suspension. All of the polished specimens were cleaned with acetone in an ultrasonic water bath for 5 min prior to microstructure observations.

Dumbbell-shaped Co-Cr alloy specimens of the four groups (*n* = 6), meeting dimensional requirements for ISO specification 22674 [9,11,16], were fabricated for the mechanical properties evaluation by a tensile test in the same way as the disc-shaped specimens. In the SLM group, the longitudinal direction (i.e., the tensile direction) of the dumbbell specimens was fixed parallel to the building direction [14].

### 4.2. XRD Analysis

For a specimen from each group, phase identification was performed by X-ray diffractometry (XRD) (MAXima_X XRD-7000, Shimadzu Corp., Kyoto, Japan) with an accelerating voltage of 30 kV, a beam current of 30 mA, a 2θ angle scan range of 30° to 100°, a scanning speed of 2°/min, a sampling pitch of 0.02°, and a preset time of 0.6 s.

### 4.3. Microscopic Characterization

The microstructures of the disc specimens of each group were observed using optical microscopy (OM) analysis (MM-40/2U, Nikon, Tokyo, Japan). For microstructural characterizations and elemental composition analyses, the disc specimens of each group were also examined by field emission-scanning electron microscopy (FE-SEM, JSM-6700F, Jeol, Tokyo, Japan) with energy dispersive X-ray spectroscopy (EDS, Oxford Instruments, Abingdon, UK) under an accelerating voltage of 15 kV. A backscattered electron (BSE) detector was used to obtain the atomic number, Z-contrast images, or orientation (or crystallographic) contrast image of the grain and subgrain microstructures [33]. Elemental point analysis and elemental area mapping analysis were carried out to determine the composition of various characteristic sites of the surfaces.

To determine the crystallographic orientation, EBSD scans were performed on a FE-SEM equipped with the Nordlys Max EBSD detector (Oxford Instruments) under accelerating voltage of 20 kV [34]. A step size of 0.1 μm was used in a hexagonal scan grid. Measured points with confidence indices less than 0.1 were eliminated to reduce inaccuracy in EBSD measurements and analysis. These points are depicted in black in the constructed EBSD maps [34].

### 4.4. Mechanical Properties

The six dumbbell-shaped specimens for each alloy were loaded in tension at a crosshead speed of 1.5 mm/min using a universal testing machine with an extensometer (Model 3366, Instron Inc., Canton, MA, USA), according to ISO specification 22674 [16]. Values of ultimate tensile strength, 0.2% yield strength, percent elongation after fracture, and Young’s modulus were obtained with the aid of the universal testing machine software. One fractured surface for each alloy was observed using a FE-SEM.

### 4.5. Statistical Analysis

After first checking for normal distribution and equal variances (Shapiro-Wilk and Levene tests, respectively), the results of the mechanical properties were statistically compared using one-way ANOVA and Tukey’s multiple comparison test at *α* = 0.05. The yield strength data were log_10_ transformed to meet homogeneity of variance prior to analysis. The statistical analyses were carried out using SPSS 17.0 for Windows (SPSS Inc., Chicago, IL, USA).

## 5. Conclusions

Within the limitations of this in vitro study, the following conclusions can be drawn:

- Co-Cr dental alloys prepared via three new CAD/CAM-based processing techniques (milling (ML), selective laser melting (SLM), and milling/post-sintering (ML/PS)) showed differences in microstructure and related mechanical properties not only from the traditional casting technique, but also among each other.
- Overall, the four techniques can be ranked as follows based on the mechanical properties evaluated in this study (in decreasing order): SLM, ML/PS, casting (CS), and ML.

## Figures and Tables

**Table 1:** Brand names, manufacturing methods, manufacturers, and elemental compositions of the four Co-Cr alloys tested.

Brand Name	Manufacturing Method (Group Code)	Manufacturer	Elemental Composition (wt %) *							
			Co	Cr	W	Nb	V	Mo	Si	Fe
StarLoy C	Casting (CS)	DeguDent, Hanau-Wolfgang, Germany	59.4	24.5	10	2	2	1	1	0.1
Magnum Lucens	Milling (ML)	MESA di Sala Giacomo and C. S.n.c, Travagliato, Italy	63	28	3	4	N/A	<1	1	<1
Remanium^®^ star CL	Selective laser melting (SLM)	Dentaurum GmbH and Co. KG, Ispringen, Germany	60.5	28	9	<1	N/A	N/A	1.5	<1
Soft Metal™	Milling/post-sintering (ML/PS)	LHK, Chilgok, Korea	63	29	N/A	N/A	N/A	6	<1	N/A

* As provided by the manufacturers. N/A: not available.

**Table 2:** Results of quantitative elemental point analysis (see Figure 5).

Group	Point	Element (wt %)								
		Co	Cr	W	Nb	V	Mo	Si	Mn	C
(a) CS	1	65.36	23.50	7.41	0.84	1.65	0.82	0.42	-	-
	2	54.03	22.01	13.92	4.06	1.50	1.82	0.79	-	1.87
	3	49.34	15.68	17.78	9.74	0.93	2.78	1.66	-	2.09
(b) ML	1	65.23	31.63	1.67	0.86	-	-	0.61	-	-
	2	50.80	14.98	3.98	27.70	-	-	2.54	-	-
	3	34.60	13.43	3.16	46.04	-	-	2.77	-	-
(c) SLM	1	71.33	22.56	4.70	-	-	-	0.82	0.58	-.
(d) ML/PS	1	67.12	26.20	-	-	-	4.68	0.60	-	1.41
	2	17.16	66.64	-	-	-	9.39	-	-	6.80
